# Severe celiac trunk stenosis following blunt trauma in a patient with diabetic ketoacidosis: a diagnostic dilemma of traumatic injury versus median arcuate ligament syndrome

**DOI:** 10.3389/fmed.2026.1884767

**Published:** 2026-08-13

**Authors:** Ke-er Huang, Qi-yuan Zhou, Lei Wang, Yi-ping Feng, Qiang Li

**Affiliations:** 1 Department of Emergency Medicine, Second Affiliated Hospital Zhejiang University School of Medicine, Hangzhou, China; 2 Department of Nursing, Second Affiliated Hospital Zhejiang University School of Medicine, Hangzhou, China

**Keywords:** blunt trauma, celiac trunk stenosis, diabetic ketoacidosis, median arcuate ligament syndrome, visceral vascular injury

## Abstract

**Introduction:**

Severe proximal celiac trunk stenosis or occlusion following blunt trauma is exceedingly rare, with potential etiologies including acute traumatic vascular injury, thrombosis, and underlying median arcuate ligament syndrome (MALS). In patients complicated by type 1 diabetic ketoacidosis (DKA), overlapping symptoms such as nausea and vomiting may arise from both metabolic derangement and visceral hypoperfusion, frequently obscuring the underlying vascular pathology and complicating diagnostic decision-making.

**Methods:**

We report the case of a 33-year-old man with poorly controlled type 1 diabetes mellitus who sustained multiple blunt injuries after being crushed by an approximately 400-kg object, resulting in sternal, rib, and pelvic fractures with hemoperitoneum. He developed persistent nausea, vomiting, and hyperglycemia, and was initially diagnosed with DKA. Despite correction of metabolic abnormalities with insulin therapy, fluid resuscitation, and acid–base balance, gastrointestinal symptoms persisted, prompting dedicated abdominal contrast-enhanced computed tomography (CT) and CT angiography (CTA).

**Results:**

Imaging revealed severe proximal celiac trunk stenosis (approximately 80% luminal narrowing) with near-occlusion, post-stenotic dilatation, a fish-hook configuration, and extensive collateral circulation, without definite intimal flap, thrombus, or atherosclerotic plaque. These findings were suggestive of pre-existing MALS with trauma-induced decompensation, although acute traumatic injury could not be entirely excluded. Following multidisciplinary evaluation, conservative management – comprising glycemic control, fluid resuscitation, antiemetic therapy, and nutritional support – was pursued given hemodynamic stability, preserved collateral perfusion, and absence of end-organ ischemia. Gastrointestinal symptoms resolved completely by hospital day 7. The patient subsequently underwent successful closed reduction and internal fixation of pelvic fractures and was discharged on postoperative day 3 without vascular intervention.

**Conclusion:**

His case underscores the diagnostic challenge posed by concurrent DKA and suspected MALS in the setting of blunt trauma. Celiac trunk dysfunction should be considered in patients with high-energy trauma who experience persistent nausea or vomiting after metabolic correction, even without hemodynamic instability or overt abdominal signs. Early contrast-enhanced CT/CTA is essential for timely diagnosis, and in carefully selected patients with adequate collateral circulation and no evidence of ischemia, individualized conservative management can be a safe and effective strategy. Multidisciplinary collaboration among trauma, vascular, endocrinology, and radiology teams is crucial for optimal management of such complex cases.

## 1 Introduction

Celiac trunk stenosis or occlusion is a rare but potentially life-threatening visceral vascular disorder. Common etiologies include atherosclerosis, thrombosis, and median arcuate ligament syndrome (MALS). In contrast, blunt trauma-related celiac trunk injury is exceedingly uncommon, accounting for less than 0.1% of all visceral vascular injuries because of the deep retroperitoneal location of the celiac trunk, its protection by the rib cage and surrounding organs, and its limited mobility (1, 2). Nevertheless, once injury occurs, it may rapidly result in foregut hypoperfusion, hepatic ischemia, or even hemorrhagic shock. Owing to the extensive mesenteric collateral circulation, some patients initially present only with nonspecific symptoms such as nausea, vomiting, or abdominal discomfort, which may delay or obscure the diagnosis (3, 4).

MALS results from chronic extrinsic compression of the proximal celiac trunk by a low-lying median arcuate ligament (MAL) (5). Although asymptomatic celiac artery compression is relatively common in the general population, trauma may precipitate decompensation of previously compensated stenosis, leading to acute symptom onset. Meanwhile, type 1 diabetic ketoacidosis (DKA) can cause severe dehydration, hypovolemia, hypercoagulability, and gastrointestinal dysmotility. Symptoms including nausea, vomiting, and abdominal pain substantially overlap with those caused by celiac trunk hypoperfusion, thereby further complicating recognition of an underlying vascular etiology (6, 7). To date, no established diagnostic or therapeutic guidelines exist for trauma-related celiac trunk stenosis or occlusion, particularly in patients concurrently presenting with DKA and suspected MALS.

Here, we report a patient with multiple blunt traumatic injuries complicated by type 1 diabetes mellitus-related DKA whose imaging studies demonstrated severe proximal celiac trunk stenosis with imaging features suggestive of MALS. The exact etiology remained uncertain and may have involved traumatic vascular injury, underlying MALS, or both. We focus on the clinical presentation, diagnostic challenges, and successful conservative management strategy. This case highlights the complex interaction between chronic vascular compromise and acute triggering factors. To our knowledge, reports describing the coexistence of blunt trauma, DKA, and suspected MALS remain exceedingly rare. In addition, we provide a comprehensive review of the literature on trauma-related celiac trunk dysfunction to facilitate multidisciplinary management of similarly complex cases.

## 2 Case presentation

A 33-year-old man was admitted with chest and lower back pain 2 days after a crush injury. On July 7, 2025, while at work, his thoracoabdominal region was compressed by an approximately 400-kg object, resulting in persistent chest and lumbar pain accompanied by limited mobility, chest tightness, and dyspnea. During hospitalization at a local hospital on the day of injury, he developed nausea and vomiting of gastric contents. Symptoms transiently improved after antiemetic therapy (specific medications unknown) but recurred after treatment discontinuation. The referring hospital diagnosed pelvic fracture, sternal fracture, multiple rib fractures, mediastinal hematoma, and closed pneumothorax. He was transferred to our emergency department on July 9, 2025. His medical history included an 8-year history of type 1 diabetes mellitus managed with subcutaneous insulin therapy, although glycemic control had been poor. He denied any prior history of recurrent postprandial abdominal pain, chronic nausea or vomiting, weight loss, or previous abdominal trauma.

Because the patient was transferred from another province without complete medical records or original imaging, a repeat trauma assessment was performed upon admission. On admission, the patient complained of chest and back pain associated with persistent nausea and vomiting. Vital signs were as follows: temperature, 36.7 °C; blood pressure, 123/72 mmHg; respiratory rate, 15 breaths/min; heart rate, 111 beats/min; and Glasgow Coma Scale score, 15. Physical examination revealed chest wall tenderness and a questionable positive chest compression test. The abdomen was soft without tenderness or rebound tenderness, and no costovertebral angle tenderness was present. Muscle strength and tone in all extremities were normal, although pigmentation was observed in both lower limbs. Pelvic compression testing was negative.

**FIGURE 1:**
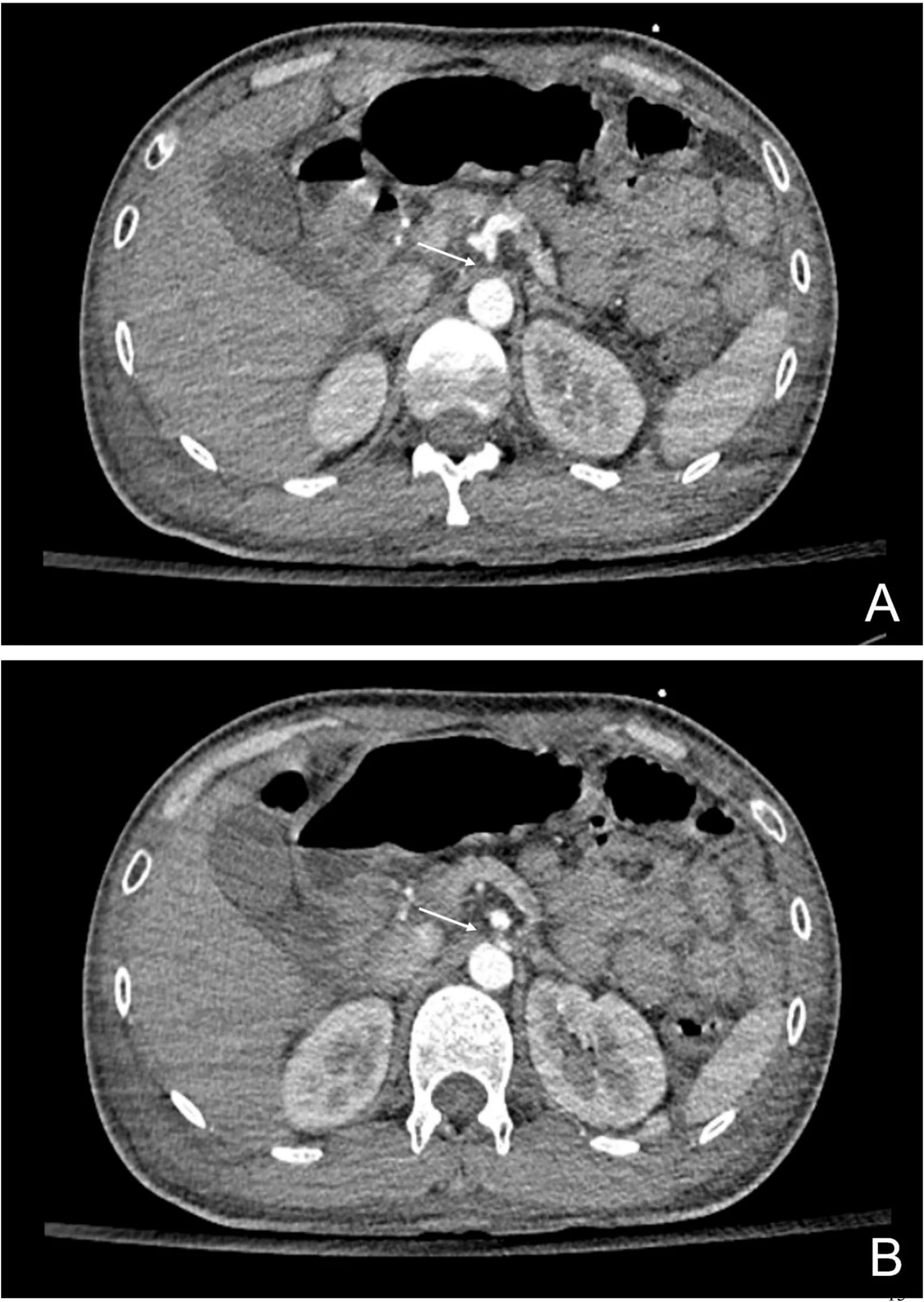
**(A, B)** Results of chest enhanced CT scan. Review of the chest enhanced CT showed that the proximal celiac trunk was markedly stenosed at the time of admission, but this finding was not reported.

Repeat trauma CT demonstrated sternal fracture, multiple rib fractures (left ribs 3–9 and right ribs 4–8), left transverse process fractures of L1–L3, multiple pelvic fractures, hemoperitoneum, and mesenteric and omental fat stranding without definite solid-organ injury. Contrast-enhanced chest CT further revealed a retrosternal hematoma, bilateral pulmonary contusions with atelectasis, and small bilateral pleural effusions. Cranial and cervical spine CT showed no abnormalities. Retrospective review of the admission contrast-enhanced chest CT revealed stenosis at the origin of the celiac trunk that had not been recognized in the original radiology report (Figure 1).

Laboratory findings included a blood glucose level of 19.30 mmol/L, hemoglobin of 61 g/L, red blood cell count of 2.01 × 10^12^/L, and whole-blood lactate of 1.20 mmol/L. The patient initially received analgesics, omeprazole, metoclopramide, supportive trauma care, and insulin therapy; however, hyperglycemia and gastrointestinal symptoms persisted. On hospital day 2, fasting blood glucose increased to 22.0 mmol/L. Arterial blood gas analysis showed pH 7.276, bicarbonate 12.9 mmol/L, base excess −11.2 mmol/L, urinary ketones 2 mmol/L, and β-hydroxybutyrate 7.02 mmol/L, consistent with mild-to-moderate DKA. Following endocrinology consultation, aggressive intravenous fluid resuscitation, electrolyte replacement, and a basal-bolus insulin regimen (insulin glargine plus insulin lispro) were initiated. Gastroenterology consultation was also obtained because of persistent nausea and vomiting.

**FIGURE 2:**
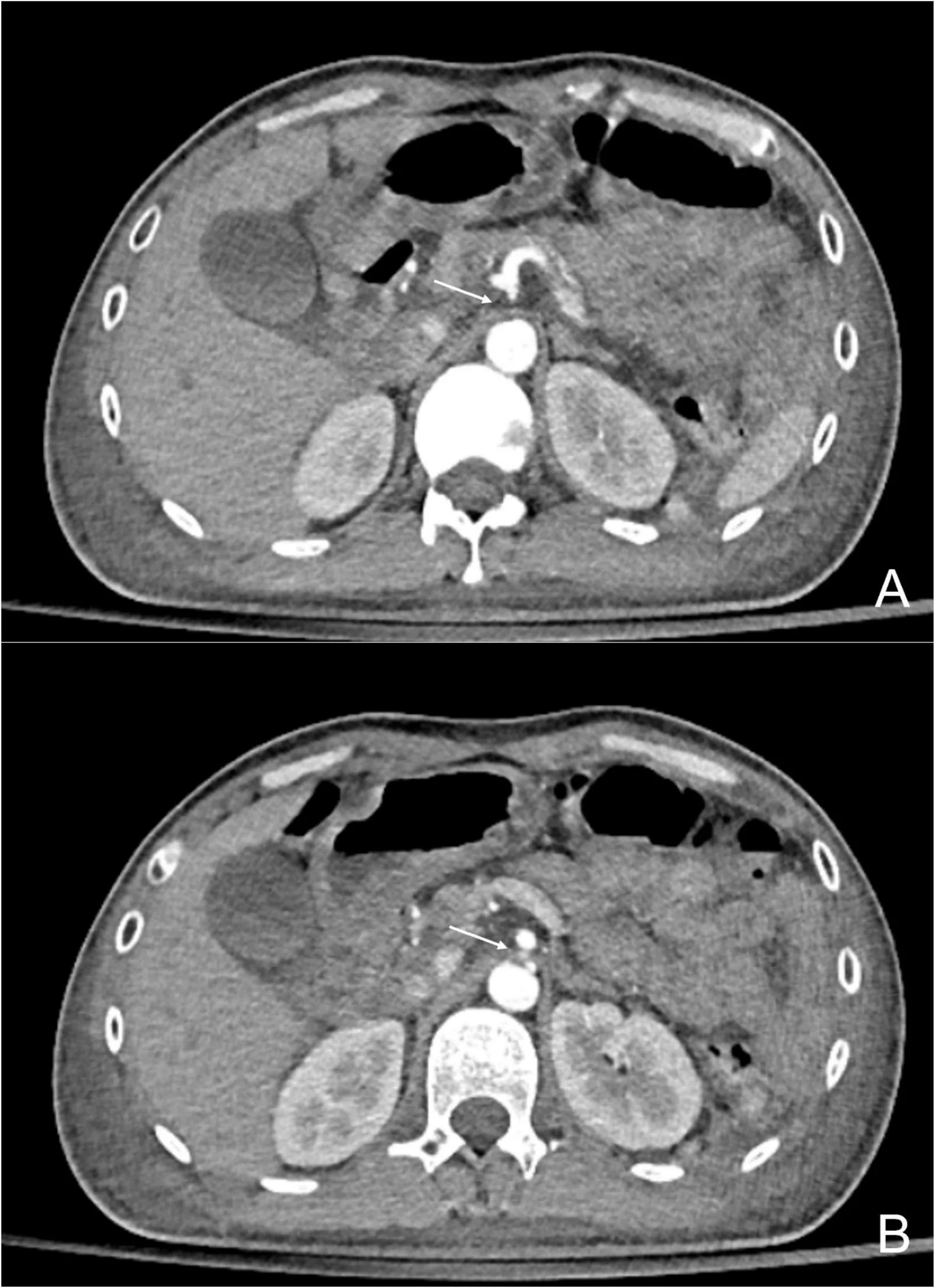
**(A, B)** Results of full abdominal enhanced CT scan. The proximal part of the celiac artery was narrowed and occluded. Abdominal and pelvic hemorrhage and effusion, swelling of the anterior sacral soft tissue and the bilateral lumbar muscles, and slight swelling and thickening of some small intestines and intestinal walls.

**FIGURE 3:**
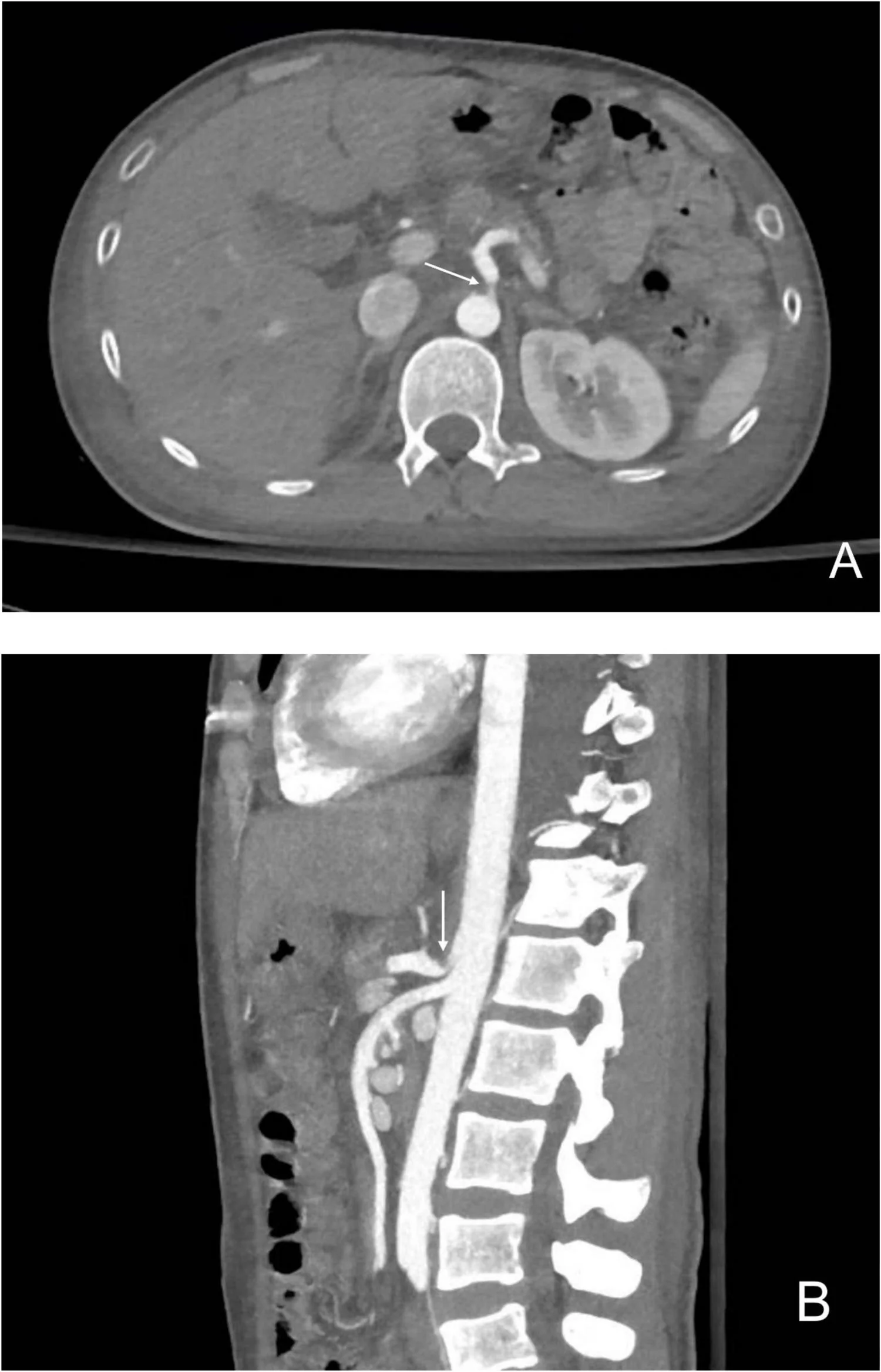
**(A, B)** Results of abdominal CT angiography. Repeated abdominal CT angiography demonstrated high-grade stenosis of the proximal celiac trunk, obvious fish-hook configuration and post-stenotic dilatation, although the median arcuate ligament could not be adequately assessed because of limited image quality.

**FIGURE 4:**
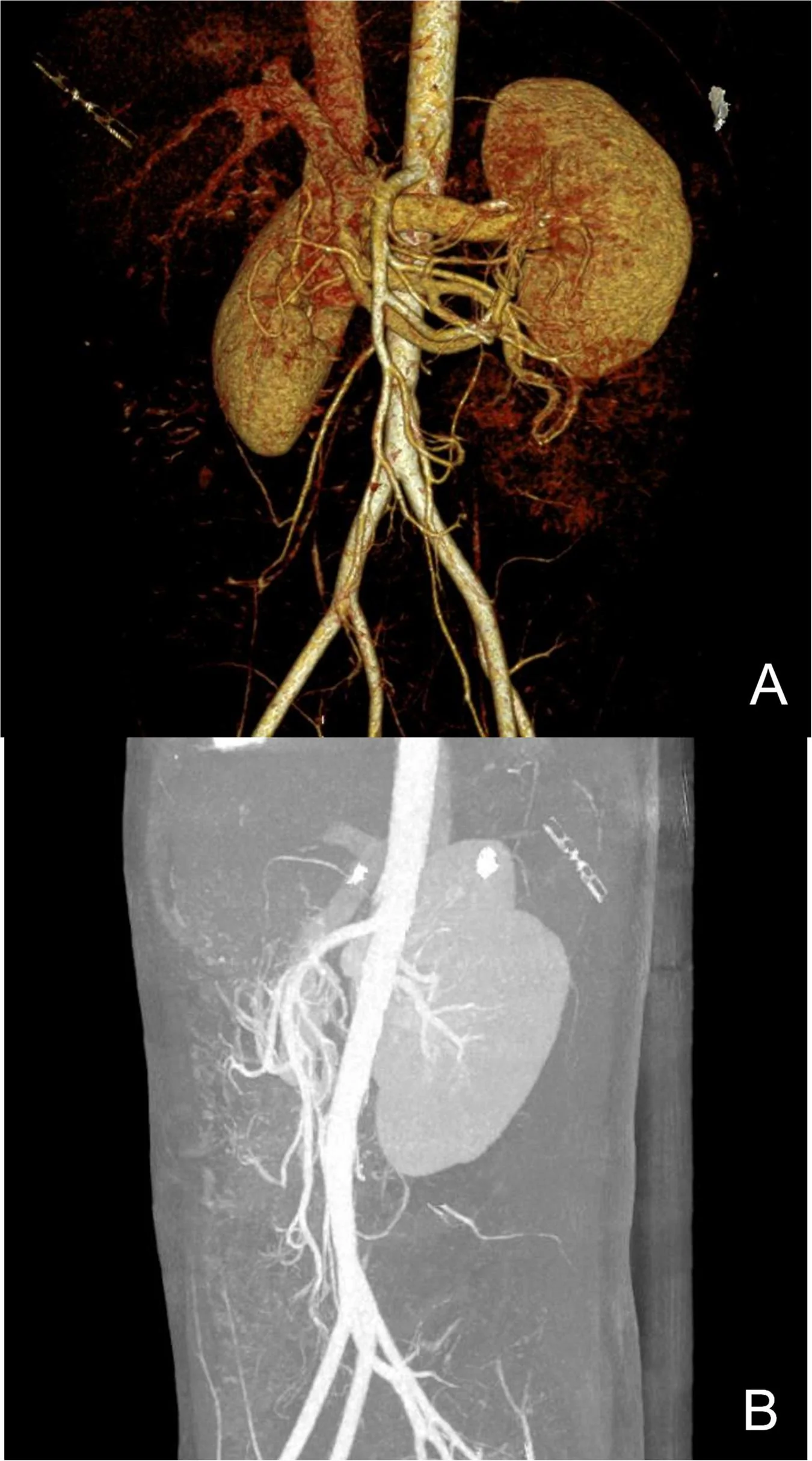
**(A, B)** Technical artifacts in three-dimensional CTA reconstruction of abdominal vessels. The image is degraded by extensive red flocculent artifacts, resulting in poor delineation of vessel margins and obscuration of key anatomical structures, including the origin of the celiac trunk.

By hospital day 3, after 24 h of stable glucose control, arterial pH had normalized (>7.4), blood glucose fluctuated between 6.1 and 10.5 mmol/L, electrolytes normalized, and liver function remained within normal limits. However, despite treatment with metoclopramide and ondansetron, nausea and vomiting persisted. Abdominal examination remained unremarkable, and both endocrinology and gastroenterology consultations concluded that hyperglycemia or electrolyte imbalance alone could not fully explain the symptoms. Persistent gastrointestinal symptoms after DKA resolution prompted further abdominal imaging. Contrast-enhanced abdominal CT demonstrated segmental thickening of portions of the small bowel and colon wall, along with severe stenosis at the origin of the celiac trunk (Figure 2). Repeat abdominal CT angiography performed on hospital day 5 again demonstrated severe proximal celiac trunk stenosis (approximately 80% luminal narrowing) with near-occlusion but preserved distal perfusion through extensive collateral circulation (Figure 3). Imaging revealed no definite intimal flap, thrombus, or atherosclerotic plaque. On post-discharge re-review with the radiology department, the CTA was reassessed and demonstrated post-stenotic dilatation and a characteristic fish-hook configuration suggestive of MALS, although visualization of the median arcuate ligament itself was limited because of image quality. Because of the severity of the patient’s injuries, the quality of three-dimensional reconstruction images was limited, precluding clear visualization of typical MAL compression findings (Figure 4).

Following multidisciplinary evaluation by the general and vascular surgery teams, conservative management was recommended because the patient remained hemodynamically stable, had adequate collateral circulation, and showed no evidence of end-organ ischemia. Repeat vascular assessment on hospital day 5 confirmed persistent severe stenosis without interval progression, and surgical or endovascular intervention was not recommended. By hospital day 7, nausea and vomiting had markedly improved, allowing resumption of a diabetic diet. On the following day, gastrointestinal symptoms had completely resolved despite a transient increase in blood glucose. Packed red blood cell transfusion was performed before orthopedic surgery to optimize hemoglobin levels.

**FIGURE 5:**
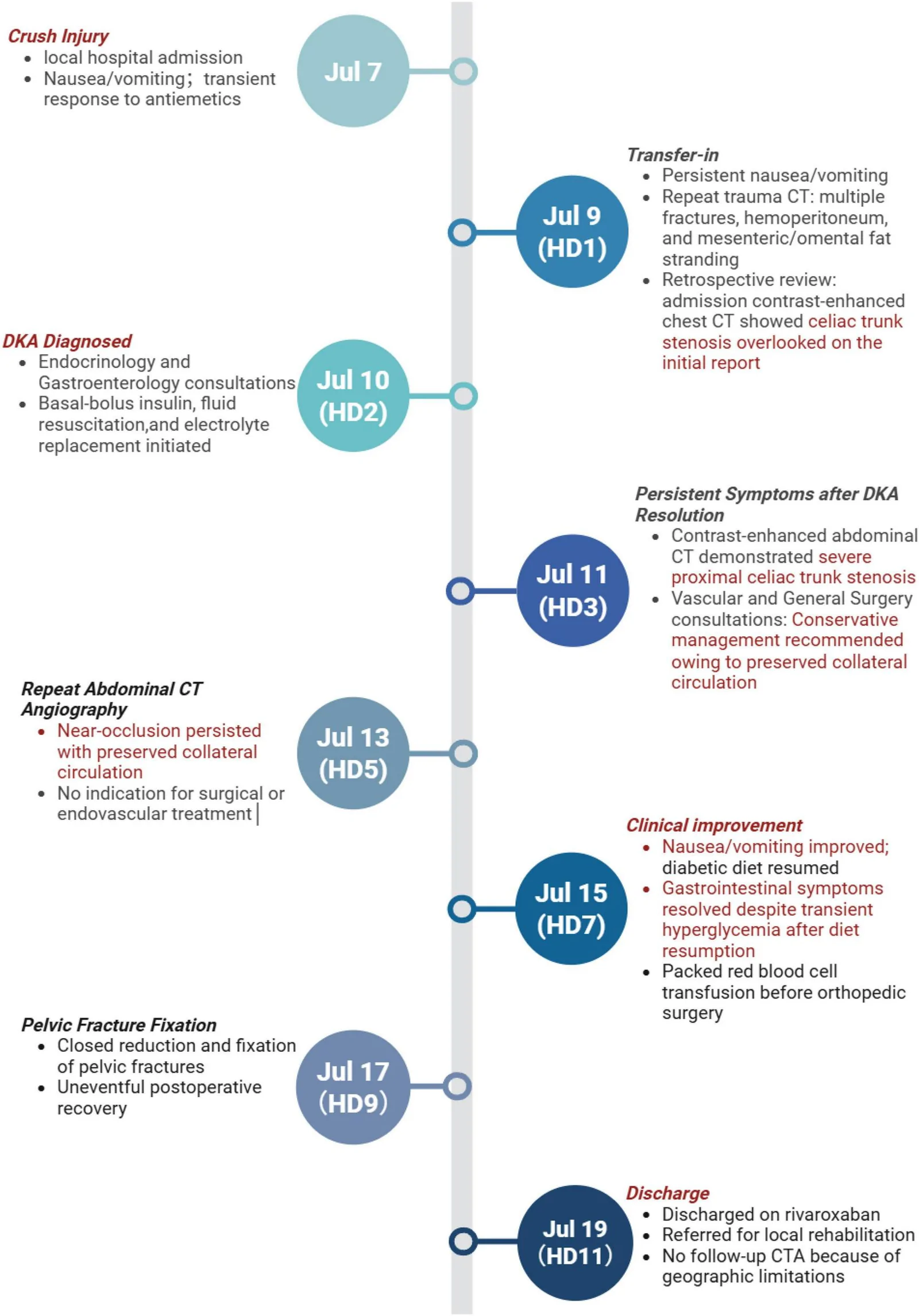
Timeline.

The patient also had multiple displaced pelvic fractures involving the right sacrum, ilium, superior and inferior pubic rami, and left pubis. Orthopedic consultation recommended surgical fixation after correction of DKA and anemia. Because of the risks of occult bleeding associated with acute trauma and pelvic fractures, pharmacologic anticoagulation was temporarily withheld, and mechanical thromboprophylaxis, serial coagulation monitoring, and lower-extremity ultrasonography were performed while red blood cell transfusion and intravenous iron supplementation were administered. Because hemoglobin had stabilized after transfusion, pharmacologic thromboprophylaxis was reconsidered. This was further supported by a marked increase in D-dimer levels and the very high risk of venous thromboembolism associated with pelvic fracture surgery. On hospital day 9, after stabilization of hemoglobin levels and complete resolution of gastrointestinal symptoms, the patient underwent closed reduction and internal fixation with external fixation of the right pelvic fractures. Intraoperative blood loss was approximately 10 mL. Postoperatively, anticoagulation with enoxaparin (0.2 mL once daily) was initiated along with fluid therapy, analgesia, and insulin treatment. Recovery was uneventful, and the patient was discharged on postoperative day 3 with rivaroxaban 10 mg once daily. Because the patient elected to continue rehabilitation in his hometown and immediate hospitalization at a local institution could not be guaranteed, oral rivaroxaban was prescribed at discharge to facilitate continued thromboprophylaxis. He was referred for local rehabilitation. Owing to geographic limitations, follow-up CTA was not performed after discharge, preventing assessment of dynamic changes in celiac trunk stenosis or potential chronic MALS-related findings. During manuscript revision, attempts were made to re-contact the patient; however, he could not be reached. Family members reported satisfactory recovery without recurrent nausea or vomiting during subsequent rehabilitation, although no follow-up imaging was available. The clinical course and key events are summarized in Figure 5.

## 3 Discussion and literature review

This case represents an exceptionally rare clinical scenario involving severe proximal celiac trunk stenosis following multiple blunt traumatic injuries, complicated by DKA and radiological features suggestive of MALS. Rather than establishing a new disease entity, this case highlights the diagnostic complexity created by the coexistence of possible chronic vascular compression, acute trauma, and metabolic disturbance. Although the available imaging findings, including a characteristic fish-hook configuration, post-stenotic dilatation, and extensive collateral circulation, are most compatible with pre-existing MALS, trauma-induced symptomatic decompensation represents a plausible explanation for the acute presentation. However, the exact contribution of chronic compression, trauma-related vascular changes, and other systemic factors cannot be definitively determined. In particular, persistent nausea and vomiting occurred in the setting of multiple competing explanations, including DKA, severe trauma, anemia, medication exposure, and potential celiac trunk-related foregut hypoperfusion. Therefore, this case emphasizes the importance of integrating radiological findings, clinical evolution, and exclusion of alternative causes rather than attributing symptoms solely to vascular abnormalities. Despite the diagnostic uncertainty, conservative management was selected based on the patient’s hemodynamic stability, preserved collateral perfusion, and absence of end-organ ischemia; this illustrates that in carefully selected patients, a non-interventional approach may be appropriate even when the exact etiology remains unresolved (8).

The unique anatomical characteristics of the celiac trunk confer relative resistance to routine abdominal trauma. The vessel is located deep within the retroperitoneum, protected by the rib cage and cushioned by surrounding structures including the stomach, greater omentum, and pancreas (9, 10). In addition, the celiac trunk is relatively short, and its origin is fixed to the spine by connective tissue and surrounding fat, limiting vascular mobility and requiring substantial kinetic energy to produce injury (11–13). Consequently, traumatic celiac artery injury accounts for only a very small proportion of reported visceral vascular injuries, and isolated blunt traumatic occlusion is exceedingly rare (14). However, these same anatomical features may predispose the vessel to chronic shear stress and extrinsic compression, thereby contributing to the development of atherosclerotic stenosis, dissection, aneurysm formation, and MALS (15). MALS results from extrinsic compression of the proximal celiac trunk by a low-lying median arcuate ligament. Most individuals with such compression remain clinically compensated through well-developed collateral circulation between the superior mesenteric and celiac arterial systems (16). Symptomatic MALS typically presents with postprandial abdominal pain, nausea, vomiting, early satiety, and weight loss, although the clinical course is highly variable. Although no universally accepted diagnostic gold standard exists, CTA remains the principal imaging modality in routine clinical practice, whereas expiratory-phase CTA and Doppler ultrasonography with respiratory maneuvers provide additional diagnostic confidence by demonstrating the dynamic nature of vascular compression during inspiration and expiration. Typical radiological features include focal proximal hook-shaped (“fish-hook”) stenosis, post-stenotic dilatation, and respiratory variation in the degree of stenosis (17).

Increasing evidence suggests that blunt trauma and MALS may not represent entirely independent entities. Several reports have described patients in whom trauma appeared to precipitate symptomatic deterioration of previously asymptomatic celiac artery compression (18). Proposed mechanisms include trauma-related diaphragmatic displacement, transient hemodynamic alterations, vasospasm, inflammatory changes, and reduced compensatory reserve in patients with chronic stenosis. Accordingly, trauma-related celiac trunk dysfunction may represent a pathological spectrum ranging from acute vascular injury to trauma-induced decompensation of previously compensated MALS. Nevertheless, current evidence is derived almost exclusively from isolated case reports and small case series, and the relative contribution of acute traumatic injury versus pre-existing celiac artery compression often remains uncertain.

To better characterize trauma-associated celiac trunk dysfunction, we systematically reviewed the published literature and identified 26 reports describing 30 individual patients between 1964 and 2025 from PubMed^®^ and Web of Science (Table 1). Unlike previous summaries, the reported cases were classified according to the predominant vascular mechanism described by the original authors, including traumatic intimal injury (dissection-related lesions), acute thrombosis/occlusion without definite dissection, complete vascular disruption (transection/avulsion), and trauma-associated pre-existing or suspected MALS/extrinsic celiac compression.

**TABLE 1:** Published reports of blunt trauma-associated celiac trunk dysfunction categorized according to the predominant vascular etiology (30 patients from 26 reports).

Etiology category	Ref	Age/sex	Traumatic mechanism	Type of injury	Symptoms	Diagnostic method	Management	Prognosis
Group A: Traumatic intimal injury (dissection-related lesions)	(25)	41/M	MVC	Dissection (intimal tear)	Flank pain, acute epigastric pain	Contrast-enhanced CT, angiography	Endovascular stenting	Recovery
	(26)	26/M	High fall	Dissection with 85% stenosis (intimal tear)	Asymptomatic, transient elevation of liver function	CT angiography	Conservative (antiplatelet, BP control)	Recovery
	(27)	44/F	MVC	Dissection (intimal tear)	Epigastric pain, transient elevation of liver function	Contrast-enhanced CT	Conservative (anticoagulation, BP control)	Recovery
	(28)	68/M	MVC	Occlusion (thrombosis secondary to dissection)	Progressive hypotension, lactic acidosis	Abdominal CT, exploratory laparotomy	Antiplatelet therapy (failed)	Death
	(29)	18/M	MVC	Stenosis (caused by dissection)	Delayed liver failure	CT angiography, angiography	Multiviscular endovascular stenting	Recovery
	(30)	18/M	Fall injury	Dissection (intimal tear)	Worsening abdominal pain	CT angiography	Conservative observation (anticoagulation contraindicated)	Recovery (lifelong follow-up)
	(31)	15/M	MVC	Occlusion (caused by dissection)	Progressive abdominal pain, hypotension	CT angiography	Endovascular stenting	Recovery (mid-term patency)
	(32)	29/M	High fall	Dissection + pseudoaneurysm (intimal tear)	Not specified in the article	Whole-body contrast-enhanced CT	Covered stenting	Recovery
	(33)	61/M	MVC	Dissection with stenosis (intimal tear)	No abdominal pain	Whole-body contrast-enhanced CT	Conservative (antiplatelet)	Recovery
	(34)	45/M	MVC	Dissection (intimal tear) + splenic infarction	Delayed abdominal pain	Whole-body contrast-enhanced CT	Anticoagulation attempted (discontinued)	Stable at discharge
Subtotal	10 reports	10 patients (33%)						
Group B: Acute thrombosis/occlusion	(1)	15/F	High fall	Acute occlusion (suspected thrombosis)	Epigastric pain, abnormal liver function	Contrast-enhanced CT	Conservative supportive care	Recovery
	(14)*a*	19/M;	MVC	Occlusion (thrombosis)	Dyspnea	Contrast-enhanced CT, interventional angiography	Trans-SMA embolization + anticoagulation;	Recovery
	(14)*b*	37/M	High fall	Occlusion (thrombosis)	Abdominal distension, nausea	Contrast-enhanced CT, CTA	Conservative (anticoagulation)	
	(35)	19/F	MVC	Splenic injury + pre-existing celiac artery occlusion	Abdominal pain, hemorrhagic shock	Abdominal contrast-enhanced CT	Splenic artery embolization via collateral pathway	Recovery
Subtotal	3 reports	4 patients (13%)						
Group C: Complete vascular disruption	(36)	17/M	MVC	Avulsion (complete vascular transection)	Hemorrhagic shock	Contrast-enhanced CT, exploratory laparotomy	Open ligation	Recovery
	(37)	39/M	Heavy object crush	Avulsion + pseudoaneurysm (vascular transection)	Not specified	Contrast-enhanced CT, angiography	Endovascular repair (stenting + branch embolization)	Recovery
	(38)	47/M	High fall	Avulsion + pseudoaneurysm (vascular transection)	Asymptomatic	CT angiography	Conservative observation	Recovery
	(39)	75/M	MVC	Avulsion (complete vascular transection)	Hemorrhagic shock	CT angiography, angiography	Celiac trunk ligation + aorto-celiac trunk bypass	Survived with complications, died at 22 months
	(40)	58/M	MVC	Avulsion (complete vascular transection)	Delayed hypotension	CT angiography	Open suture ligation	Recovery
	(41)	54/M	MVC	Avulsion (complete vascular transection)	Delayed hypoxia, hypotension	Whole-body contrast-enhanced CT	Balloon occlusion + open suture ligation	Recovery
	(42)	25/M	MVC	Hepatic proper artery transection (vascular transection)	Initially stable, then rapidly deteriorated	Contrast-enhanced CT, E-FAST	Exploratory laparotomy + ligation	Death
Subtotal	7 reports	7 patients (23%)						
Group D. Pre-existing or suspected MALS/extrinsic celiac compression associated with trauma	(18)*a*	17–37/F (One of 3F)	Fall	60%-99% stenosis (MALS-induced)	Abdominal pain, nausea, weight loss, feeding intolerance	Ultrasonography, angiography, diagnostic celiac plexus block	MAL division + extensive perivascular neurolysis + celiac ganglionectomy	Recovery
	(18)*b*	17-37/F (One of 3F)	Postoperative	60%-99% stenosis (MALS-induced)	Abdominal pain, nausea, weight loss, feeding intolerance	Ultrasonography, angiography, diagnostic celiac plexus block	MAL division + extensive perivascular neurolysis + celiac ganglionectomy	Recovery
	(18)*d*	17-37/F (One of 3F)	Pregnancy-related	60%-99% stenosis (MALS-induced)	Abdominal pain, nausea, weight loss, feeding intolerance	Ultrasonography, angiography, diagnostic celiac plexus block	MAL division + extensive perivascular neurolysis + celiac ganglionectomy	Recovery
	(18)*d*	M (Age n/s)	Not specified (part of series)	60%-99% stenosis (MALS-induced)	Abdominal pain, nausea, weight loss, feeding intolerance	Ultrasonography, angiography, diagnostic celiac plexus block	MAL division + extensive perivascular neurolysis + celiac ganglionectomy	Recovery
	(43)	37/M	MVC	Dissection/hemorrhage (secondary to suspected MALS)	Hemorrhagic shock	Contrast-enhanced CT, angiography	Retrograde embolization + aortic covered stenting	Recovery
	(44)	30/M	Blunt abdominal trauma	Dissection-induced occlusion (secondary to chronic MALS)	Hemorrhagic shock, delayed abdominal pain	CT angiography	Pseudoaneurysm embolization	Recovery
	(45)	18/M	MVC	Acute occlusion (on chronic MALS background)	Asymptomatic	Contrast-enhanced CT, angiography	Conservative (antiplatelet, BP control)	Spontaneous recanalization
	(46)	18/F	MVC	Stenosis (external compression by spinal dislocation)	Mild abdominal pain, back pain	X-ray, contrast-enhanced CT, angiography	Spinal fixation (indirect decompression)	Recovery
	(47)	36/M	MVC	Celiac trunk occlusion, MALS-induced SMA syndrome	Hepatic and splenic hypoperfusion, liver dysfunction, lactic acidosis	Abdominal CT, mesenteric angiography	Conservative treatment	Recovery
Subtotal	6 reports	9 patients (30%)						
Grand total	26 reports	30 patients						

Cases were categorized according to the predominant pathological mechanism described by the original authors. Because overlap between chronic MALS, traumatic dissection, thrombosis, and secondary occlusion may exist in individual patients, some cases could reasonably fit more than one category. Classification was therefore intended to facilitate comparison rather than imply mutually exclusive disease entities. CTA, computed tomography angiography; MALS, median arcuate ligament syndrome; MVC, motor vehicle collision; SMA, superior mesenteric artery; BP, blood pressure; LFT, liver function tests; n/s, not specified.

Traumatic intimal injury represented the largest subgroup (10/30, 33%), followed closely by trauma-associated MALS/extrinsic compression (9/30, 30%), complete vascular disruption (7/30, 23%), and isolated thrombosis or occlusion (4/30, 13%). Most patients were young or middle-aged men who sustained high-energy blunt trauma, predominantly motor vehicle collisions, followed by falls from height and crush injuries. Despite considerable heterogeneity in vascular pathology, preserved collateral circulation was consistently associated with favorable outcomes, whereas complete vascular disruption and progressive visceral ischemia accounted for most reported deaths. The available literature also highlights an important diagnostic limitation. In most published cases, pre-trauma vascular imaging, dedicated expiratory-phase CTA, or Doppler ultrasonography with respiratory maneuvers were unavailable, making it difficult to distinguish pre-existing asymptomatic MALS from acute traumatic vascular injury or trauma-induced decompensation of chronic celiac compression. Accordingly, the precise contribution of each mechanism remains uncertain in many patients and should be interpreted in conjunction with the mechanism of injury, serial imaging findings, clinical evolution, and evidence of chronic vascular remodeling, rather than inferred solely from the temporal association with trauma.

Diagnosis of celiac trunk stenosis in the acute trauma setting is particularly challenging because clinical manifestations are often nonspecific. Approximately half of the reported patients presented with abdominal pain, whereas symptoms such as nausea, vomiting, epigastric discomfort, and abdominal pain may overlap with manifestations caused by trauma itself, metabolic disturbances, medication effects, or gastrointestinal injury. Furthermore, asymptomatic celiac trunk compression is relatively common, and subtle vascular abnormalities may be overlooked during emergency imaging assessments primarily focused on immediately life-threatening injuries. In our literature review, nearly one-quarter of reported patients were initially asymptomatic, likely reflecting the protective effect of collateral circulation. CTA remains the primary diagnostic modality because it simultaneously evaluates vascular patency, organ perfusion, and associated traumatic injuries. Imaging findings suggestive of traumatic vascular injury include intimal flap formation, mural thrombus, pseudoaneurysm, active contrast extravasation, or abrupt vascular occlusion. In contrast, findings favoring MALS include focal hook-shaped stenosis, post-stenotic dilatation, respiratory variation, and the absence of intraluminal injury. Doppler ultrasonography with respiratory maneuvers and dedicated expiratory-phase CTA may further improve diagnostic accuracy. Complete vascular transection represents the most severe injury pattern and is typically associated with active hemorrhage, hemorrhagic shock, and a high mortality rate. Among the 30 reported cases, only four patients died from hemorrhagic shock and multiorgan failure, whereas the remaining patients achieved favorable outcomes. Nevertheless, blunt trauma-related celiac trunk stenosis continues to carry substantial mortality risk if not promptly recognized and treated (19), and refractory gastrointestinal symptoms may significantly worsen patient prognosis (20, 21).

In the present case, the available imaging findings favored pre-existing MALS with trauma-induced symptomatic decompensation over isolated acute traumatic vascular injury, although the latter could not be completely excluded. Retrospective review of admission contrast-enhanced chest CT, obtained for thoracic trauma assessment, demonstrated stenosis at the origin of the celiac trunk that had not been recognized in the original radiology report, indicating that the lesion was already present before dedicated abdominal vascular imaging. Subsequent abdominal CTA demonstrated approximately 80% luminal narrowing with post-stenotic dilatation, a characteristic fish-hook configuration, and extensive collateral circulation, without evidence of intimal flap, mural thrombus, pseudoaneurysm, active contrast extravasation, or significant atherosclerosis. Collectively, these findings are more consistent with chronic extrinsic compression than isolated acute vascular injury.

Nevertheless, trauma likely contributed to symptom onset. Blunt thoracoabdominal trauma may alter diaphragmatic mechanics, transiently increase compression of the celiac origin, provoke vasospasm, or reduce the compensatory reserve of chronic collateral circulation, thereby converting previously asymptomatic celiac artery compression into a clinically significant lesion. Because no pre-trauma imaging was available, however, the relative contribution of chronic MALS and acute trauma remains uncertain. The diagnostic process also deserves consideration. At presentation, persistent nausea and vomiting could reasonably be attributed to multiple concurrent conditions, including DKA, severe anemia, traumatic pain, retrosternal hematoma, mesenteric injury, bowel wall edema, medication effects, and occult bleeding. In addition, liver function remained normal and there was no clinical or radiological evidence of visceral ischemia, making clinically significant celiac trunk dysfunction less likely during the initial trauma evaluation. Only after correction of metabolic acidosis and persistent gastrointestinal symptoms despite supportive therapy was dedicated abdominal vascular imaging performed, leading to recognition of the vascular abnormality. In retrospect, careful re-evaluation of the admission trauma CT was crucial for reconstructing the temporal evolution of the lesion

Several diagnostic limitations should also be acknowledged. Dedicated expiratory-phase CTA and Doppler ultrasonography with respiratory maneuvers were not performed because acute trauma, pain, and limited patient cooperation made dynamic vascular assessment impractical. Although these examinations could have strengthened the diagnosis of MALS, routine CTA remains the principal first-line imaging modality in clinical practice, and post-discharge multidisciplinary review with radiologists identified post-stenotic dilatation and a fish-hook configuration highly suggestive of chronic MAL compression. Likewise, no pre-trauma imaging or follow-up vascular studies were available because the patient lived in another province and could not be re-contacted. Therefore, MALS cannot be definitively confirmed, and the final diagnosis should be interpreted as radiologically suspected MALS with unresolved contribution from trauma-related vascular changes.

Another major diagnostic challenge in this case was the coexistence of type 1 DKA. DKA was most likely precipitated by major blunt trauma together with physiological stress, pain, reduced oral intake, and pre-existing poor glycemic control, rather than by celiac trunk stenosis itself, and thus represented an important confounding factor rather than a direct consequence of celiac trunk dysfunction. Once DKA developed, however, differentiating metabolic from non-metabolic causes of nausea and vomiting became difficult. DKA, severe trauma, anemia, bowel edema, analgesic medications, and occult abdominal injury may all produce similar gastrointestinal manifestations (22). The persistence of vomiting despite rapid correction of acidosis, normalization of electrolyte balance, and satisfactory glycemic control prompted reconsideration of alternative diagnoses. Furthermore, gastrointestinal symptoms resolved under continued conservative management despite a transient rebound in blood glucose, suggesting that DKA alone could not fully explain the clinical course. Previous reports have described recurrent DKA in patients with symptomatic MALS (23, 24). Unlike those cases, however, our patient had no history of chronic postprandial pain, weight loss, or recurrent vomiting before trauma. Therefore, the novelty of this report lies not in the coexistence of MALS and DKA itself, but in the complex diagnostic scenario in which trauma-induced DKA complicated the interpretation of persistent gastrointestinal symptoms in a patient with radiologically suspected celiac trunk dysfunction.

Management should be individualized according to hemodynamic status, collateral perfusion, lesion characteristics, and evidence of end-organ ischemia (14). Patients without visceral ischemia may be managed conservatively with fluid resuscitation, metabolic correction, nutritional support, symptom control, and close clinical observation, whereas progressive ischemia, hemorrhage, or failed conservative treatment may require endovascular or surgical intervention.

In our patient, preserved collateral circulation, stable liver function, absence of bowel or solid-organ ischemia, and substantial bleeding risk from multiple pelvic fractures favored conservative management. Pharmacologic anticoagulation was initially withheld because of the high risk of occult traumatic bleeding. After pelvic fixation, stabilization of hemoglobin, and increasing venous thromboembolism risk, thromboprophylaxis with enoxaparin was initiated. At discharge, rivaroxaban was selected because the patient chose rehabilitation in another province, where immediate hospitalization and continued injectable anticoagulation could not be guaranteed, making oral therapy more practical for maintaining thromboprophylaxis. Gradual symptom resolution with supportive treatment alone supports the feasibility of conservative management in carefully selected patients with preserved collateral circulation

## 4 Limitations

Several limitations should be acknowledged. First, this is a single-center case report, which limits the generalizability of the findings. In addition, multiple pelvic fractures and the complex traumatic background may have obscured potential vascular-related signs, making it difficult to completely distinguish certain clinical manifestations from skeletal or soft tissue injuries. Second, because of limited three-dimensional reconstruction quality and the absence of dedicated expiratory-phase CTA, high-resolution Doppler ultrasonography with respiratory maneuvers, dynamic digital subtraction angiography (DSA), and histopathological evidence, MALS could not be definitively confirmed. Although the CTA demonstrated post-stenotic dilatation and fish-hook configuration suggestive of MALS, visualization of the median arcuate ligament itself was limited because of image quality. Dedicated duplex ultrasound with respiratory maneuvers, the standard test for confirming MALS, was not performed during admission because of the patient’s multiple pelvic fractures, extensive injuries, and abdominal wall tenderness. Consequently, the relative contributions of traumatic vascular injury and underlying MALS remain uncertain, though MALS with trauma-induced decompensation appears most plausible. Third, repeat vascular imaging was not performed after symptom resolution, and standardized long-term imaging follow-up could not be completed because of geographic limitations. During manuscript revision, attempts were made to re-contact the patient; however, he could not be reached. Family members reported satisfactory recovery without recurrent nausea or vomiting during subsequent rehabilitation, although no follow-up imaging was available. Therefore, further assessment of vascular morphological changes, collateral circulation evolution, and the long-term durability of conservative management was not possible. Finally, the relative contributions of DKA, trauma-related physiological stress, multiple trauma-related factors (anemia, pain, opioids, ileus, bowel wall edema, bleeding), and visceral hypoperfusion to the patient’s gastrointestinal symptoms could not be fully quantified.

## 5 Conclusion

We report an exceptionally rare case of severe proximal celiac trunk stenosis following multiple blunt traumatic injuries complicated by type 1 DKA and radiological findings suggestive of MALS. Rather than suggesting that trauma caused a purely acute vascular injury, this case illustrates that trauma may precipitate symptomatic decompensation of previously compensated stenosis. The imaging findings – including extensive collateral circulation, post-stenotic dilatation, and fish-hook configuration – support pre-existing MALS as the most likely underlying etiology, although the contribution of trauma-related vascular changes cannot be completely excluded.

Because DKA and celiac trunk hypoperfusion share substantial overlap in clinical manifestations, clinicians should maintain a high index of suspicion for underlying visceral vascular pathology in patients with high-energy blunt trauma who continue to experience persistent nausea, vomiting, or abdominal discomfort despite correction of metabolic abnormalities, even in the absence of hemodynamic instability or overt abdominal signs. Careful re-evaluation of the initial trauma CT and selective CTA when clinically indicated are critical for timely diagnosis.

This case further demonstrates that individualized conservative management may represent a safe and effective strategy in patients with preserved collateral circulation and no evidence of end-organ ischemia. Moreover, multidisciplinary collaboration among trauma surgeons, vascular surgeons, endocrinologists, and radiologists is essential for the early recognition and optimal management of such complex patients.

## Data Availability

The raw data supporting the conclusions of this article will be made available by the authors, without undue reservation.
